#  

**DOI:** 10.1111/jdi.14324

**Published:** 2024-12-04

The publication of invaluable papers in the Journal of Diabetes Investigation depends on the prompt, careful review of submitted manuscripts. We would like to thank the Editorial Board members, the International Editorial Board members, the Assistant Editorial Board members and the following experts for reviewing manuscripts from September 1, 2023 to August 31, 2024.

Mohammed S. Abusamaan

Adegbenga B. Ademolu

Houssein Ahmadi

Zubair Ahmed

Ken‐Ichi Aihara

Yoichi Ajiro

Satoru Akazawa

Hamed Akbari

Gulali Aktas

Nurshad Ali

Shigeru Aoki

Tomohisa Aoyama

Keiko Arai

Atsushi Araki

Osamu Arisaka

Shunichiro Asahara

Kenji Ashida

Yoshimasa Aso

Hande Atalay

Ahmad Khusairi Azemi

Kengo Azushima

Masayuki Baba

Asaad Ma Babker

Mohamed F. Balaha

Ryotaro Bouchi

David S. Boyer

Ali Cetin

Chi‐Ming Chan

Te‐Fu Chan

Min‐Cheol Chang

Deqing Chen

Deyan Chen

Harn‐Shen Chen

Juncao Chen

Ming‐Wei Chen

Yang Ching Chen

Yen‐Lin Chen

Yi‐Rong Chen

Kenneth Cheng

Kyu Yong Cho

Soo Choi

Chih‐Hsun Chu

Daisuke Chujo

Seung Min Chung

Dana Mihaela Ciobanu

Flávia Campos Corgosinho

Andre Luiz Costa

Xiaona Cui

Makoto Daimon

Taura Daisuke

Undurti Das

Takahisa Deguchi

Masashi Demura

Wuquan Deng

Sunil Deshpande

Domenico Di Raimondo

Yu Ding

Kentaro Doi

Jocelyn J. Drinkwater

Joao M. N. Duarte

Tuba Duman

Jun Eguchi

Akira Endo

Kang‐Chih Fan

Lei Feng

Gabor Ferneisz

Chia‐Po Fu

Toshihito Fujii

Rumi Fujikawa

Junji Fujikura

Shimpei Fujimoto

Shiho Fujisaka

Yoshihito Fujita

Yukihiro Fujita

Yoshio Fujitani

Michiaki Fukui

Masato Furuhashi

Shinya Furukawa

Hiroto Furuta

Yechiel N. Gellman

Stacey Gorniak

Atsushi Goto

Weaam Gouda

Alpesh Goyal

Tanja Groten

Xuejiang Gu

Yunjuan Gu

Keyu Guo

Kyoung Hwa Ha

Masahide Hamaguchi

Akihiro Hamasaki

Toshiaki Hanafusa

Rikinari Hanayama

Shinichi Harashima

Goji Hasegawa

Koshi Hashimoto

Yoshitaka Hashimoto

Nabil A. Hasona

Yuji Hataya

Yoshitaka Hayashi

Yasuaki Hayashino

Sakoda Hideyuki

Shinji Higuchi

Tatsuhito Himeno

Tsutomu Hirano

Satoshi Hirayama

Takahisa Hirose

Inoue Hiroshi

Yushi Hirota

Takanori Honda

Minha Hong

Ruby Hoo

Ichiro Horie

Takeshi Horii

Yukio Horikawa

Masayuki Hosoi

Yi‐Ting Hsieh

Paul Hsu

Cheng Hu

Haofei Hu

Chia‐Luen Huang

Chuiguo Huang

Cn Huang

Yu‐Tung Huang

Zhimin Huang

Mikael S Huhtala

Sartaj Hussain

You‐Cheol Hwang

Yasuo Ido

Masaki Igarashi

Katsumi Iizuka

Kaori Ikeda

Eri Ikeguchi

Junta Imai

Fumiaki Imamura

Minako Imamura

Yasushi Ishigaki

Hideki Ishii

Takahiro Ishikawa

Masato Iwabu

Katsuomi Iwakura

Kunihiro Iwamoto

Yoshihiko Izumida

Suriadi Jais

In Kyung Jeong

Jee‐Heon Jeong

Feng Jiang

Sang‐Man Jin

Si Jin

Hye Seung Jung

Inha Jung

N. K. Elnaggar

Naotetsu Kanamoto

Akio Kanazawa

Hideaki Kaneto

Erbil Karaman

Soji Kasayama

Masafumi Kasuga

Sayaka Katagiri

Naoto Katakami

Koichi Kato

Takehiro Kato

Yoshiro Kato

Mojtaba Kaviani

Takumi Kawaguchi

Yuji Kawaguchi

Daiji Kawanami

Eiji Kawasaki

Ben Ke

Amirmohammad Khalaji

Touch Khun

Chong Hwa Kim

Kyuho Kim

Mi Kyung Kim

So Hun Kim

Yong Kyung Kim

Tomohiko Kimura

Tadahiro Kitamura

Shigehiko Kitano

Chih‐Yuan Ko

Kazuo Kobayashi

Tatsuya Kondo

Guilan Kong

Masaya Koshizaka

Pawan Krishan

Garry Kuan

Mitsunobu Kubota

Sodai Kubota

Akihiro Kuma

Shinji Kume

Cc Kuo

Chun‐Heng Kuo

Makoto Kurano

Akio Kuroda

Yoshiki Kusunoki

C‐H Lai

Nitchakarn Laichuthai

Ivana Lapic

Davide Lauro

Yunyi Le

Eun Young Lee

I‐Te Lee

Joonyub Lee

Ting‐Wei Lee

Qifu Li

Yonghong Li

Yuanying Li

Tang Yi Liao

Chia‐Hung Lin

Kuan‐Hung Lin

Shin‐Yu Lin

Xiahong Lin

Feng Liu

Jing Liu

Ye Liu

Jingyi Lu

David Lui

Sihui Luo

Xiaoping Luo

Shuquan Lv

Yasutaka Maeda

Hiroshi Maegawa

Zhila Maghbooli

Rayaz Malik

Maria Asunción Martínez‐Brocca

Masafumi Matsuda

Munehide Matsuhisa

Michihiro Matsumoto

Takeshi Matsumura

Takashi Matsuoka

Mariia Matveeva

Shu Meguro

Catharina M. C. Mels

Runtang Meng

Takayuki Miki

Akira Mima

Hiroto Minamino

Keiichiro Mine

Koki Mise

Tomoya Mita

Junnosuke Miura

Emiri Miura‐Yura

Satoshi Miyamoto

Takeshi Miyatsuka

Hiroki Mizukami

Yifei Mo

Masoud Mohammadnezhad

Himel Mondal

Joon Ho Moon

Jun Sung Moon

Min Kyong Moon

Hiroyasu Mori

Jun Mori

Katsutaro Morino

Tomoaki Morioka

Yoshiaki Morishita

Shota Moyama

Seiichi Munesue

Chieko Murakami

Kazutoshi Murakami

Takaaki Murakami

So Nagai

Yoshio Nagai

Mototsugu Nagao

Toru Nagao

Daiji Nagayama

Gojiro Nakagami

Hiroki Nakajima

Miki Nakajima

Akinobu Nakamura

Nobuhisa Nakamura

Shuhei Nakanishi

Eitaro Nakashima

Atsuko Nakatsuka

Jakub Nalepa

Takao Nammo

Akihiko Narisada

Yuya Nishida

Akihiko Nishikimi

Rimei Nishimura

Wataru Nishimura

Daisuke Nishioka

Mitsuhiko Noda

Hiroyuki Nodera

Takashi Nomiyama

Hiroshi Nomoto

Seitaro Nomura

Shinsuke Noso

Adetunji Obadeji

Makiko Ogata

Sumito Ogawa

Masahito Ogura

Ken Ohashi

Toshiaki Ohkuma

Yasuharu Ohta

Yoichi Oikawa

Kenta Okada

Yosuke Okada

Yukiko Okazaki

Tsuyoshi Okura

Takeshi Onoue

Asuman Orhan Varoglu

Jiemin Pan

Jeong Hyun Park

Stefano Passanisi

Jinyong Peng

Cynthia Porter

Ingrid Prkacin

Mehran Rahimlou

Xingwu Ran

Hamid Riazi‐Esfahani

Hani Sabaie

Aghdas Safari

Atsuhito Saiki

Yoshifumi Saisho

Kazuhiko Sakaguchi

Naoaki Sakata

Ichiro Sakuma

Samuel Sakyi

Kazunori Sango

Shugo Sasaki

Asako Sato

Junko Sato

Tetsuhiko Sato

Jane N. T. Sattoe

Naoto Seki

Yi‐Jing Sheen

Kenichi Shikata

Michio Shimabukuro

Akira Shimada

Masashi Shimoda

Dai Shimono

Jun Shirakawa

Takuhito Shoji

Geng Song

Swayam Srivastava

Tanja Premru Srsen

Anna Stubbendorff

Ming Su

Akira Sugawara

Kenji Sugawara

Shigetaka Sugihara

Kazuhiro Sugimoto

Ken Sugimoto

Yoshihisa Sugimura

Takehiro Sugiyama

Suresh Sukumar

Atushi Suzuki

Ryo Suzuki

Kensei Taguchi

Yuji Tajiri

Matsuoka Takaaki

Mitsuyoshi Takahara

Hirokazu Takahashi

Kazuma Takahashi

Toshinari Takamura

Shin Takasawa

Takeshi Takayanagi

Masato Takeuchi

Yumi Takiyama

Yoshifumi Tamura

Jiangshan Tan

Daisuke Tanaka

Nagaaki Tanaka

Sho Tano

Yukako Tatsumi

Hiroki Teragawa

Yasuo Terauchi

Sanae Teshigawara

Nesime S. Tiskaoglu

Atsuhito Tone

Keiichi Torimoto

Masao Toyoda

Nagaoki Toyoda

Jakob Triebel

Sandra Tsoi

Shin Tsunekawa

Daisuke Tsuriya

Masaki Uchihara

Yohei Ueda

Hiroyuki Umegaki

Aditya Verma

Robert Vigersky

Masako Waguri

Kayo Waki

Chih Yuan Wang

Huabin Wang

Jun‐Sing Wang

Xinlei Wang

Yufan Wang

Junping Wen

Chung‐Ze Wu

Chunhua Wu

Hongjiang Wu

Hung‐Tsung Wu

Xifeng Wu

Zekai Wu

Weizhen Xi

Liangdi Xie

Yilian Xie

Zhenggao Xie

Chao Xu

Chengfu Xu

Jing Xu

Lingling Xu

Wenan Xu

Yaoming Xue

Kunimasa Yagi

Tetsuya Yamada

Kazuya Yamagata

Megu Yamaguchi‐Baden

Kousuke Yamahara

Tadashi Yamakawa

Mayumi Yamamoto

Takeshi Yamamoto

Yasuhiko Yamamoto

Masahiro Yamamoto

Shunsuke Yamane

Katsuya Yamazaki

Yuan‐Horng Yan

Jin Yang

Kun Yang

Wanshui Yang

Weihua Yang

Yong Yang

Hisafumi Yasuda

Tahereh Yavari

Jianping Ye

Yun‐Kai Yeh

I‐Weng Yen

Feride Taskin Yilmaz

Senay Gorucu Yilmaz

Jun Yin

Hisashi Yokomizo

Takashi Yoneda

Hideto Yonekura

Mark Yorek

Yoko Yoshida

Satoshi Yoshiji

Keiji Yoshioka

Hongzhao You

Bin Yu

Cheng Yu

Mengyue Yu

Xuefeng Yu

Yongfu Yu

Yu Yuan

Jae‐Seung Yun

Bo Zhang

Huabing Zhang

Meilin Zhang

Mingliang Zhang

Sen Zhang

Xue‐Lian Zhang

Yan Zhang

Haiming Zhao

Xiaolong Zhao

Jian Zhou

Yang Zou

